# Cue-Elicited Anxiety and Alcohol Craving as Indicators of the Validity of ALCO-VR Software: A Virtual Reality Study

**DOI:** 10.3390/jcm8081153

**Published:** 2019-08-02

**Authors:** Alexandra Ghiţă, Olga Hernández-Serrano, Yolanda Fernández-Ruiz, Miquel Monras, Lluisa Ortega, Silvia Mondon, Lidia Teixidor, Antoni Gual, Bruno Porras-García, Marta Ferrer-García, José Gutiérrez-Maldonado

**Affiliations:** 1 Department of Clinical Psychology and Psychobiology, University of Barcelona, Passeig de la Vall d’ Hebron 171, 08035 Barcelona, Spain; 2 Department of Physical Therapy, EUSES University of Girona, 17190 Salt, Spain; 3 Addictive Behaviors Unit, Hospital Clinic of Barcelona, 08036 Barcelona, Spain

**Keywords:** ALCO-VR, virtual reality, cue-exposure, alcohol use disorder, alcohol craving, anxiety, social drinkers

## Abstract

Background: This study is part of a larger project aiming to develop a virtual reality (VR) software to be implemented as a clinical tool for patients diagnosed with alcohol use disorder (AUD). The study is based on previous research in which we identified factors that elicit craving for alcohol in a sample of AUD patients, and which led to the development of a virtual reality software to be used in cue exposure treatments of alcohol use disorder (ALCO-VR). The main objective of this study was to test the effectiveness of ALCO-VR to elicit cue-induced craving and anxiety responses among social drinkers (SD) and AUD patients. Our secondary objective was to explore which responses (cue-induced craving or anxiety) can best differentiate between AUD patients and the SD group. Method: Twenty-seven individuals (13 AUD patients and 14 SD) participated in this study after giving written informed consent. Their anxiety and alcohol craving levels were measured by different instruments at different stages of the procedure. The VR equipment consisted of Oculus Rift technology, and the software consisted of the ALCO-VR platform. Results: Our data indicate that the ALCO-VR software can elicit responses of anxiety and alcohol craving, especially in the group of AUD patients. The cue-induced anxiety response differentiated AUD patients and the SD group better than the cue-induced craving response. Conclusions: The general interest in applying new technologies to the assessment and treatment of mental health disorders has led to the development of immersive real-life simulations based on the advantages of VR technology. Our study concluded that the ALCO-VR software can elicit anxiety and craving responses and that cue-induced anxiety responses can distinguish between AUD and SD groups better than cue-induced craving. The data on craving and anxiety were assessed consistently by different instruments. In addition, we consider that ALCO-VR is able to ecologically assess cue-induced anxiety and alcohol craving levels during exposure to VR alcohol-related environments.

## 1. Introduction

Alcohol misuse is considered to be a serious condition with important negative consequences at a personal and societal level [1,2,3,4]. Numerous studies have emphasized that excessive alcohol use may establish a solid basis for general heavy drinking patterns, binge-drinking episodes, and further development of alcohol use disorder (AUD) in adulthood [5,6,7,8]. In AUD, a substantial number of patients respond to treatment by accomplishing individual goals such as controlled drinking or total abstinence [9]. However, many individuals struggle with maintaining long-term abstinence; despite treatment, they experience relapses, especially in the first three months [10,11]. AUD is a complex disorder and many factors contribute to its development and maintenance. The current study focuses on the interplay between anxiety and alcohol craving as factors interfering in long-term abstinence.

Anxiety, ranging from momentary distress to a psychopathological diagnosis, is common in the majority of patients with AUD, and is considered a facilitating factor in the onset of alcohol consumption [12]. Anxiety is involved not only in the pre-phase of drinking behaviors, but is also a predominant symptom experienced during alcohol withdrawal in patients with AUD [13]. A growing and solid body of research has focused on understanding the phenomena underlying anxiety disorders and AUD, since individuals with dual diagnoses are more vulnerable in terms of recovery and maintaining abstinence [14,15,16]. A network modeling analysis revealed that anxiety-related states such as social anxiety and stress are directly engaged in craving elicitation in patients diagnosed with AUD [14]; thus, a strong causal relationship was established between anxiety and craving for alcohol, which further precipitates drinking behaviors and, implicitly, relapse. This new model explains the critical involvement of this causative relationship between anxiety and alcohol craving in patients diagnosed with AUD.

Alcohol craving, described in the literature as a “pathological appetite”, is an unbearable desire to use alcohol [17] and has a crucial role in alcohol misuse behaviors [18]. Explained by the classical conditioning theory [19], alcohol craving is acquired through repetitive drinking behaviors accompanied by positive emotional valence [20]. Over the past two decades, laboratory research [21,22] and clinical research [23,24,25] have emphasized the strong relationship between proximity of alcohol-related stimuli that trigger alcohol craving. This context dependency theory indicates that alcohol-related stimuli gain incentive salience over time [26,27] and therefore, cues and contexts related to alcohol consumption emerge as high-risk stimuli for individuals who misuse alcohol [28]. A common method for exploring alcohol craving responses is based on the cue-exposure paradigm, which involves in vivo alcohol cue presentation, or exposure to photographic alcohol-related stimuli in non-realistic experimental or clinical settings [29]. Over the past years, an increasing interest in new technologies like virtual reality (VR) has led to the development of more ecologically valid methods to explore alcohol craving [30]. VR may add effectiveness to the classical cue-exposure paradigm by including multiple inputs like visual, auditory, olfactory or tactile stimuli, thus creating an immersive experience based on naturalistic daily-life contexts [31]. This three-dimensional (3D) system allows a high degree of interaction, which further increases the individual’s level of momentary presence within the VR environment [32]. These are fundamental variables for developing ecological momentary assessment (EMA) instruments, particularly for its use in clinical settings, as most assessment instruments in clinical psychology rely on self-reported scales [33,34]. In drug cue reactivity, VR has been included in many protocols, primarily studying craving for alcohol [35,36], tobacco [37,38,39], methamphetamine [40] or cocaine [41]. In AUD, VR has also been implemented in the treatment of patients with the final aim of reducing craving levels and, implicitly, of preventing further relapses. A comprehensive review of the existing studies indicated that all studies focused on the applications of VR as a therapy tool showed consistent results in terms of craving reduction, thus favoring the inclusion of this technology in the treatment of substance use disorders [30].

The present study is based on the results of a previous study, in which we identified triggering factors for alcohol craving, aiming to develop significant VR alcohol-related environments. Our main objective was to validate the ALCO-VR platform (a virtual reality software to be used in cue exposure treatments of alcohol use disorder) and to test its effectiveness in terms of the elicitation of cue-induced alcohol craving and anxiety responses in a sample of social drinkers (SD) and patients diagnosed with AUD. A secondary objective of our study was to explore which cue-elicited response (alcohol craving or anxiety) differentiates better between AUD patients and SD. We expected to obtain significant differences between the neutral VR condition and the VR alcohol-related environments regarding cue-induced anxiety and alcohol craving responses in the two groups, AUD patients and SD. Similarly, we expected to find statistically significant differences between AUD and SD groups in terms of their alcohol craving and anxiety responses. Based on an earlier study in bulimia nervosa [42], we expected cue-induced anxiety responses to differentiate between AUD patients and SD better than cue-induced alcohol craving responses. 

## 2. Method

### 2.1. Participants

Twenty-seven individuals participated in this study, 13 patients from the Addictive Behaviors Unit, Hospital Clinic of Barcelona and 14 SD, students at the University of Barcelona. Ethical approval was obtained from the Ethics Committees at the University of Barcelona and Hospital Clinic of Barcelona. All patients and students participated in this study after providing written informed consent. The clinical sample consisted of 13 outpatients, 8 men and 5 women (M_age_ = 48, SD = 4.8), diagnosed with AUD according to the *Diagnostic and Statistical Manual of Mental Disorders* (5th ed.) [43]. Patients presented comorbid diagnoses of borderline personality disorder, anxiety, and attention deficit hyperactivity disorder (ADHD). Their pharmacotherapy included anxiolytic, antidepressant, disulfiram, and antipsychotic medication. Seven patients reported occasional tobacco, cannabis, and cocaine use in the month prior to the experiment, but none reported alcohol consumption in the last month as they were under treatment for AUD at Hospital Clinic of Barcelona. The mean abstinence period was 68 days, ranging from one month to one year. Self-reports of substance use and abstinence data were supported by results of urine analyses performed in all patients. The inclusion criteria were having an AUD diagnosis and receiving outpatient treatment for AUD. Individuals with comorbid disorders like personality disorders or anxiety disorders and occasional use of illicit drugs (e.g., cannabis or cocaine) or tobacco were included, but those with severe comorbid psychopathology (e.g., psychosis or dementia), severe cognitive impairment that might interfere with the task completion, or anti-craving medication (e.g., naltrexone) were excluded. Pregnant women were also excluded.

The control group consisted of 14 SD, 2 men and 12 women (M_age_ = 23, SD = 5.6), all students at the University of Barcelona. Among this group, there were self-reports of one diagnosis of depression and one diagnosis of ADHD. One student reported using anxiolytic medication, and one student antidepressant medication. Tobacco and cannabis use were self-reported by six students in the last month prior to the experiment. SD also reported their monthly consumed standard drink units (SDU). A Spanish SDU is a single consumption of 10 g of ethanol (the standard quantity of wine or beer and half the standard quantity of liquor) [44]. Students consumed approximately 9 SDU (M = 9.2, SD = 9.9) per month, ranging from 3 to 24 SDU/month. Abstinence data (M_days_ = 15, SD = 18) in this group were based on self-reports from the students. The inclusion criterion was a social drinking pattern (i.e., individuals who use alcohol on a social basis and are not identified as having a problematic pattern of alcohol use). As in the clinical group, comorbid disorders (personality disorders or anxiety) and occasional use of illicit drugs (e.g., cannabis or cocaine) or tobacco were not considered exclusion criteria. However, any participants with severe comorbid psychopathology or severe cognitive impairment were excluded.

### 2.2. Measures

Alcohol consumption was assessed with the Alcohol Use Disorder Identification Test (AUDIT) [45]. The Spanish version of AUDIT [46] is a 10-item scale assessing alcohol misuse and severity of alcohol-related problems. Responses are scored from 0 to 4 with a maximum score of 40. 

Alcohol craving was assessed with the Multidimensional Alcohol Craving Scale (MACS) [47]. The MACS is a Spanish self-reported scale aiming to assess the “intensity of alcohol craving experienced by the participant in the previous week”. Scores on each item range from 1 (“Strongly disagree”) to 5 (“Strongly agree”). There are three MACS outcome scores: “desire to drink,” “behavioral disinhibition,” and the total score, which were each graded as nonexistent, mild, moderate or intense. Moreover, a modified version of MACS (MACS-VR) was introduced in the experimental procedure to explore alcohol craving immediately after VR exposure to alcohol-related contexts and cues. The items, scores, and outcomes of the MACS-VR remained the same as in the original MACS, although the instructions were now to assess the “intensity of alcohol craving experienced during VR exposure”.

Anxiety was explored with the Spanish version of the State-Trait Anxiety Inventory (STAI) [48]. The STAI is a self-reported questionnaire with two subscales assessing how a person feels at the moment (STAI-state, state anxiety) and how a person feels in general (STAI-trait, trait anxiety). Each subscale consists of 20 items, and scores of each item range from 0 (“Not at all”) to 3 (“Very much so”). Higher scores on each subscale indicate higher levels of trait and/or state anxiety. 

Alcohol craving and anxiety experienced during VR exposure were explored with visual analogue scales (VAS). These scales are widely used as instruments to measure craving (VAS-C) and anxiety (VAS-A) during VR exposure [35]. The VAS-C is a self-reported virtual craving scale, with scores ranging from 0 to 100, where 0 is interpreted as “no craving” and 100 indicates “intense craving”. Similarly, VAS-A is a self-reported scale aiming to explore anxiety levels, with scores ranging from 0 to 100, where 0 is interpreted as “no anxiety” and 100 as “intense anxiety”. VAS-C and VAS-A are ecological scales used during exposure to VR environments.

### 2.3. Instruments

#### 2.3.1. Hardware

The VR equipment consisted of Oculus Rift head-mounted display (HMD), 1080 × 1200 resolution per eye, a 90 Hz refresh rate, and 110° field of view, sensors, touch controllers, and a computer compatible with the VR technology (INTEL(R) Core(TM) i7-2600 CPU, 16.0 GB RAM, Operating System 64 bits, processor ×64, graphic card NVIDIA GeForce GTX 1080 Ti). 

#### 2.3.2. “ALCO-VR” Software

**Figure 1:**
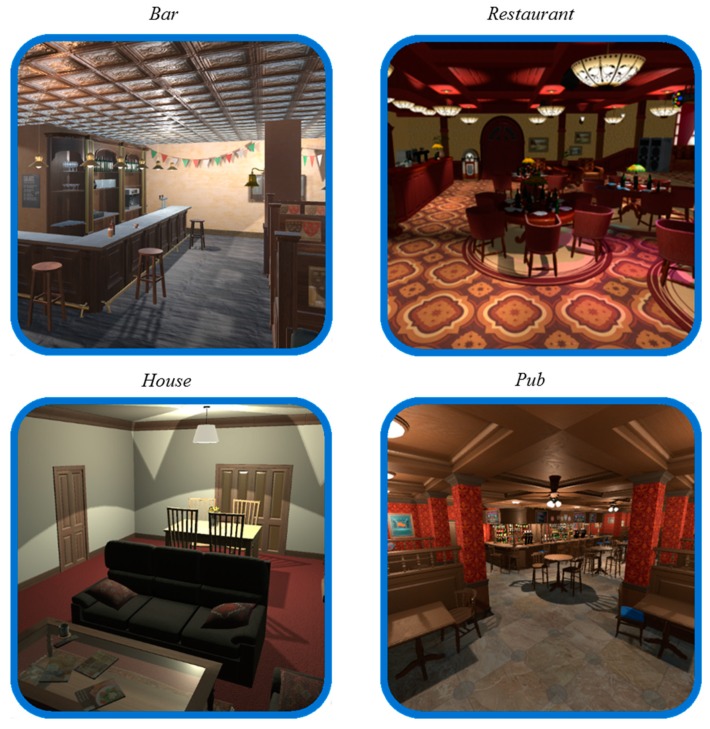
Pictures of the VR environments.

“ALCO-VR” software was developed based on the results of a previous study [49], in which we identified factors that contribute to the elicitation of alcohol craving in a sample of patients diagnosed with AUD. Based on that study, the ALCO-VR software was created, consisting of four VR alcohol-related environments: a restaurant, a bar, a pub, and at-home environments (Figure 1). These VR environments were created considering multiple variables, as in our previous research, such as social interactions (including avatars in the environments), different alcohol-related cues (a menu of 22 alcoholic drinks), or different times of day (daytime or nighttime). Hence, there were two environments during daylight (bar and restaurant) and two environments during nighttime (pub and at-home), one environment with no social interaction (the at-home environment) and three with social interaction (bar, pub, restaurant). All environments were created to simulate real-life scenarios based on patients’ experiences. The ALCO-VR platform consisted of two parts, assessment and therapy. The assessment part was the focus of the current study and the ALCO-VR created a hierarchy of exposure from the lowest rated environment with the lowest rated alcoholic drink to the highest rated environment and the highest rated alcoholic drink. On the VAS-A and VAS-C, users were asked to rate how much cue-induced anxiety and craving they considered the environments and alcoholic drinks triggered on a scale from 0 to 100. The assessment part of the ALCO-VR centered on the first five rated alcoholic beverages, the favorite drinks of each participant, which were presented in each VR environment. In addition, the software consisted of a neutral environment (a room with a white background and a glass of water), where the participants could familiarize themselves with the VR technology. A high interaction level between the user and the VR platform was considered fundamental and individuals could approach their alcoholic beverages, hold them and observe them from all angles with Oculus Touch controllers.

### 2.4. Procedure

Participants from the control group (SD group) were recruited through social media platforms at the University of Barcelona. Patients from the clinical group (AUD group) were informed about the study during one of their appointments with their psychiatrists or clinical psychologists at the Addictive Behaviors Unit at the Hospital Clinic of Barcelona. Once they had agreed to participate, they were referred to the researcher in charge of the study. Participants were asked to sign the informed consent document after a short explanatory introduction regarding the technology used. Subsequently, clinical data were collected from the patients such as dual diagnoses, medication, abstinence data, alcohol consumption, and other substance use (illicit or licit) during the month prior to the experiment. These data were confirmed by their clinical psychologist. Participants were then asked to complete the AUDIT, STAI (the trait part), and MACS. Upon completion of these self-reported scales, the researcher instructed participants how to use the touch controllers, and they were given water to drink in order to not interfere with alcohol consumption patterns. The ALCO-VR software started by assessing how much craving and anxiety was triggered by 2D images of each VR environment (pub, at home, restaurant, and bar) and each alcoholic drink on a VAS from 0 to 100. Based on these responses, the individualized exposure hierarchy consisted of the interplay between the first five chosen alcoholic beverages, and the four contexts, gradually increasing from the lowest rated drink and the lowest rated VR environment to the highest rated alcoholic beverage and highest rated environment. When this initial part of the procedure was completed, the Oculus Rift HMD was attached to the participant’s head. This represented the start of the 3D virtual experience and each participant was exposed to the VR environments following his/her hierarchy. The first instruction was to approach the beverage and observe it from all angles for approximately 20 s, but without attempting to virtually drink from it. The VR exposure started with a neutral environment, exposure to a glass of water in a white room, aiming to familiarize participants with the VR technology. This environment served as a neutral condition in the analyses and as training for the participants. The hierarchy created by the ALCO-VR platform consisted of exposure to each alcoholic drink, which appeared in each environment. Hence, participants self-reported their cue-induced alcohol craving and anxiety levels on the VAS-C and VAS-A throughout exposure to the ALCO-VR software (4 environments × 5 drinks = 20 ratings × 2 (craving and anxiety) = 40 ratings). Olfactory stimuli were introduced during the exposure procedure and corresponded to each drink. Previously prepared alcoholic beverages were transferred onto cotton pads and placed on the table, close to the participant each time a new alcoholic drink appeared during the exposure procedure. Overall, the exposure lasted for approximately 10–15 min. After the experiment, participants were asked to complete the MACS-VR, and STAI (the state part). In addition, they were also asked to rate several variables of the ALCO-VR platform on a scale from 0 to 10, with 0 considered “very low” and 10 was considered “excellent”; these variables were user-friendliness, overall quality of the software, realism of the environments, and realism of the alcoholic beverages. Finally, for the AUD group, a debriefing session was carried out at the end of the procedure with the aim of reducing momentary craving and anxiety levels and to minimize any further possible alcohol use. Each session lasted for approximately one hour. The cross-sectional sessions were delivered by an experienced clinician-scientist at VR-Psy Lab, University of Barcelona.

### 2.5. Statistical Analysis

As the assumption of normality of data was not satisfied according to the Shapiro–Wilks test *p-*value of ≤0.05, non-parametric tests were applied in this study. First, Friedman tests were run separately for each group to compare participants’ levels of craving and anxiety experienced during VR exposure to the neutral environment and to the four alcohol-related environments. Post-hoc Wilcoxon signed-rank tests were used to determine specific significant differences in craving and anxiety between the conditions in both groups. In addition, Mann–Whitney U tests were applied to explore differences between the AUD group and SD in terms of anxiety and alcohol craving levels experienced during exposure to the VR alcohol-related environments. Spearman correlations were conducted to explore the relationship between self-reported craving and anxiety responses on VAS-C/VAS-A and the results of AUDIT, STAI, and MACS. Finally, due to the gender and age imbalance, we performed analysis of covariance (ANCOVA) after converting our data into rank scores. All statistical analyses were carried out using IBM SPSS version 24.

## 3. Results

Table 1 indicates the data of the self-reported questionnaires in terms of alcohol misuse (AUDIT), trait-anxiety (STAI, trait part), alcohol craving during the last week prior to the experiment (MACS), state anxiety (STAI, state part), and momentary cue-induced alcohol craving immediately after exposure to the ALCO-VR software (MACS-VR). 

### Data of the Self-Reported Questionnaires

**Figure 2:**
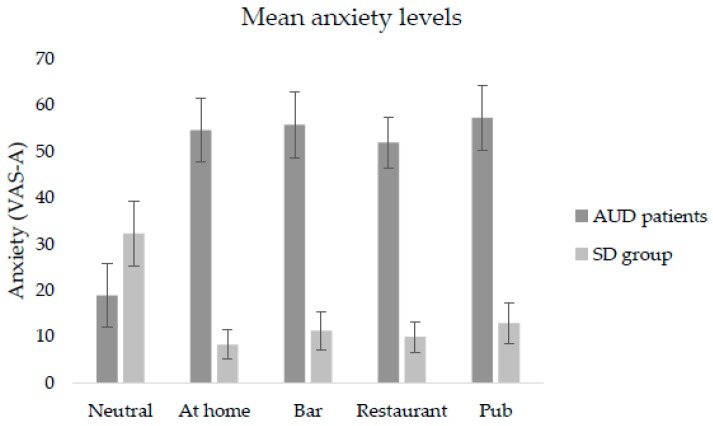
Self-reported mean levels of anxiety (A) on visual analogue scales (VAS)-A.

Significant differences were found in self-reported levels of anxiety and alcohol craving across exposure to the VR environments. The Friedman test indicated statistically significant differences in self-reported anxiety levels reported on the VAS-A across the VR environments in both AUD patients (*χ*^2^ (4) = 11.26, *p* = 0.02) and the SD group (*χ*^2^ (4) = 15.78, *p* = 0.003). In the AUD group, post-hoc Wilcoxon rank test confirmed that there were significant differences in cue-induced anxiety reported on the VAS-A between the neutral and at-home environments (*Z* = −2.621, *p* = 0.009), between neutral and bar environments (*Z* = −2.447, *p* = 0.01), between neutral and restaurant scenarios (*Z* = −2.762, *p* = 0.006) and between neutral and pub environments (*Z* = −2.691, *p* = 0.007). No statistically significant differences were found between the four VR alcohol-related environments on self-reported anxiety on the VAS-A (*p* > 0.05). Figure 2 confirms that self-reported levels of anxiety were higher during exposure to the VR alcohol-related environments than during exposure to the neutral environment. However, in the SD group, we found the opposite pattern. Although there were statistically significant differences between neutral and at-home environments (*Z* = −2.707, *p* = 0.007), between neutral and bar environments (*Z* = −2.621, *p* = 0.009), between neutral and restaurant environments (*Z* = −2.589, *p* = 0.01), and between neutral and pub environments (*Z* = −2.135, *p* = 0.03), the scores of self-reported anxiety were higher in the neutral environment than in the four VR alcohol-related environments, as Figure 2 shows.

**Figure 3:**
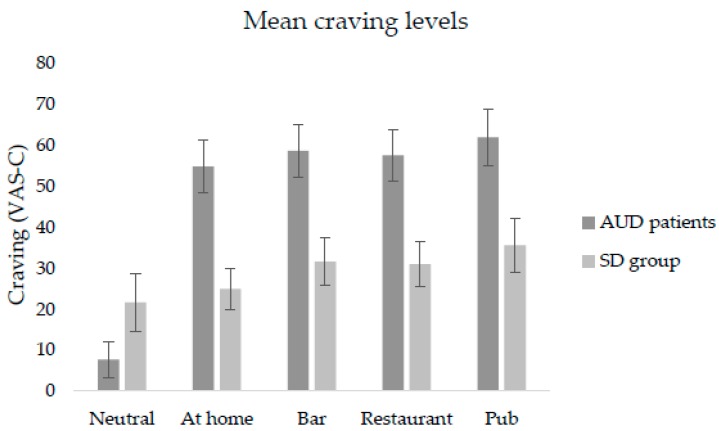
Self-reported mean levels of alcohol craving (C) on VAS-C.

Regarding self-reports of alcohol craving on the VAS-C, the Friedman test revealed a statistically significant difference across the VR environments in the AUD patients’ group (*χ*^2^ (4) = 22.83 *p* = 0.000), but not in the SD group (*p* > 0.05). In the AUD group, post-hoc analyses with Wilcoxon rank test showed significant differences in self-reported alcohol craving on the VAS-C between the neutral and at-home environments (*Z* = −3.041, *p* = 0.002), between neutral and bar environments (*Z* = −3.180, *p* = 0.001), between neutral and restaurant environments (*Z* = −3.110, *p* = 0.002), and between neutral and pub environments (*Z* = −3.110, *p* = 0.002). As can be seen in Figure 3, no statistically significant differences in alcohol craving reports on the VAS-C were found across the four VR alcohol-related environments. Both Figure 2 and Figure 3 indicate significantly higher scores in the AUD group than in the SD group regarding self-reported anxiety and alcohol craving levels on the VAS-A and VAS-C, respectively. As there were more significant differences in terms of anxiety levels compared to craving levels, we consider that cue-induced anxiety responses can better differentiate between SD and AUD patients than self-reported craving responses.

The results of the Mann–Whitney U test indicated a statistically significant difference between AUD and SD groups when comparing the mean anxiety and alcohol craving responses in each VR alcohol-related environment (*p* < 0.05). As can be seen in Table 2, self-reported anxiety and alcohol craving levels were higher in the AUD group than in the SD group across all VR alcohol-related environments. There were no statistically significant differences between AUD and SD groups regarding their anxiety and alcohol craving responses in the neutral environment (*p* > 0.05).

In addition, in the AUD group, there was a strong, positive correlation between alcohol dependence severity as shown by AUDIT and total craving reported on the VAS-C (*r_s_* = 0.783, *p* = 0.002) and total anxiety reported on the VAS-A (*r_s_* = 0.650, *p* = 0.016). In addition, there was a significant relationship between trait-anxiety as assessed by the STAI (the trait part of the questionnaire) and the total alcohol craving score reported on the VAS-C (*r_s_* = 0.667, *p* = 0.013). A positive correlation was also found between cue-induced craving as explored by the MACS-VR and total alcohol craving responses reported on the VAS-C (*r_s_* = 0.581, *p* = 0.03). No other correlations were found in anxiety and craving levels assessed by different instruments (*p* > 0.05). Regarding the SD group, the only correlations were found between cue-induced craving as assessed by MACS-VR and mean total craving reported on the VAS-C (*r_s_* = 0.629, *p* = 0.016) and mean total anxiety reported on the VAS-A (*r_s_* = 0.707, *p* = 0.005). 

Addressing the age and gender imbalance in our study, ANCOVA analyses were run to determine the effect regarding gender and age on total mean levels of anxiety and alcohol craving in SD and AUD patients. After converting the scores to ranks, we performed ANCOVA analyses on the rank scores. After adjustment for gender, there was a statistically significant group difference regarding their total mean anxiety levels reported on VAS-A *F* (1, 24) = 39.313, *p* < 0.001, partial *η*^2^ = 0.621. Post-hoc analysis was performed with a Bonferroni adjustment. Anxiety levels were significantly greater in AUD patients (M = 54.8, SE = 6.33) than the SD group (M = 10.5, SE = 3.47), a mean difference of 13.7, 95% confidence interval (CI) (9.23, 18.3), *p* < 0.001. Similarly, after adjusting for gender, there was a statistically significant group difference regarding total mean craving levels reported on VAS-C *F* (1, 24) = 10.075, *p* < 0.005, partial *η*^2^ = 0.296. Post-hoc Bonferroni analysis revealed that AUD patients (M = 58.2, SE = 6.05) reported significantly greater craving levels than the SD group (M = 30.79, SE = 5.4) (mean difference of 9.63, 95% CI (3.37, 15.90), *p* < 0.005). 

**Figure 4:**
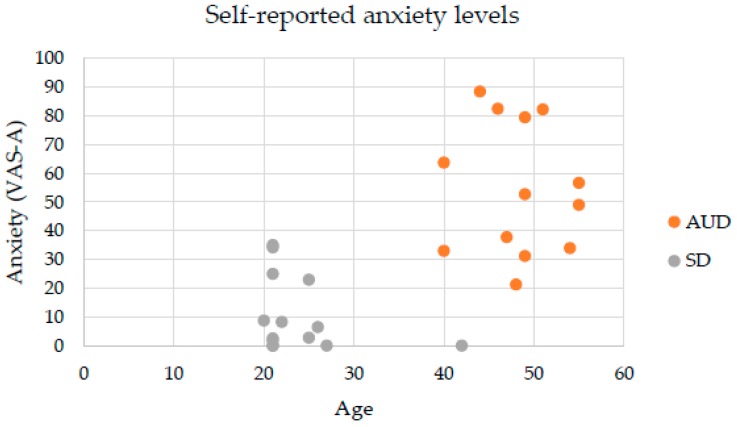
Self-reports of total mean anxiety in AUD and social drinkers (SD) groups.

**Figure 5:**
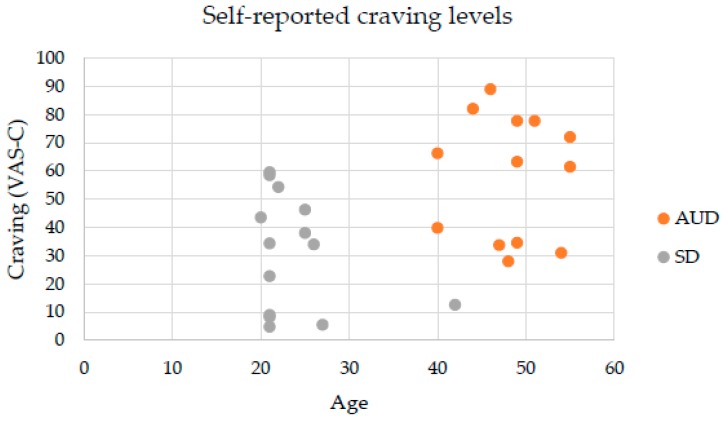
Self-reports of total mean craving in AUD and SD groups.

In addition, after adjusting for age, there were statistically significant differences between SD and AUD groups in terms of their cue-induced responses. There was a statistically significant difference between the two groups regarding their total mean anxiety levels *F* (1, 24) = 12.317, *p* < 0.005, partial *η*^2^ = 0.339. Anxiety levels were significantly greater in AUD patients (M = 48.23, SE = 6.33) compared to the SD group (M = 23, SE = 3.4) (mean difference of 17.65, 95% CI (7.27, 28.04), *p* < 0.005). Finally, there was no statistically significant difference between groups in terms of total mean craving regardless of age *F* (1, 24) = 3.764, *p* > 0.05, partial *η*^2^ = 0.136. These results can be appreciated in Figure 4 and Figure 5.

Finally, the participants rated the ALCO-VR software according to the following variables: user-friendliness (M_AUD_ = 9.15, SD = 0.89; M_SD_ = 8.93, SD = 0.91), overall quality of the software (*M_AUD_* = 8.23, SD = 1.58; M_SD_ = 8, SD = 1.3), realism of the environments (M_AUD_ = 7.77, SD = 1.53; M_SD_ = 7.8, SD = 1.5), and realism of the alcoholic beverages (M_AUD_ = 7.38, SD = 1.93; M_SD_ = 7.21, SD = 1.31).

## 4. Discussion

The current study was centered on testing the effectiveness of ALCO-VR software to induce anxiety and craving responses. Its secondary objective was to assess which craving or anxiety responses could best differentiate between the AUD and SD groups. The VR alcohol-related environments induced significantly greater anxiety levels than the neutral environment in our clinical group, but not in our control group. On the contrary, the VR alcohol-related environments induced greater craving levels than the neutral environment in both clinical and control groups. More significant alcohol craving responses were found among AUD patients.

Our results are consistent with previous research [35] and confirm the effectiveness of the ALCO-VR platform for inducing anxiety and craving responses. Patients diagnosed with AUD displayed high levels of anxiety during exposure to alcohol-related contexts compared to our control group. Furthermore, craving responses in the AUD group were even higher than in the SD group during exposure to the VR alcohol-related environments than during exposure to the neutral environment. This response pattern is supported by previous research showing greater levels of craving within alcohol-related VR environments [36,50]. In substance use disorders, VR smoking-related environments elicited higher craving levels in nicotine-dependent undergraduate students than in the control condition [51]. In a virtual reality-methamphetamine (VR-METH) cue-exposure paradigm, methamphetamine users experienced higher craving levels than in the neutral condition [40]. Additionally, in behavioral addictions, a significant internet gaming VR environment induced higher craving levels in patients diagnosed with internet gaming disorder than in a control group [52]. Similarly, in gambling disorder, frequent gamblers experienced higher craving induced by the VR environment than non-frequent gamblers [53].

Furthermore, although there were no statistically significant differences across the VR environments in our SD group, we observed a gradual increase in alcohol craving during exposure to the VR alcohol-related environments compared to the neutral scenario. We interpret this result as being representative of a group of young social drinkers, whose drinking patterns involve social interaction and peer pressure, especially in bars and pubs. The result also confirms the contextual specificity of craving for alcohol [49,54].

It is worth mentioning that both SD and AUD patients did not self-report high levels of anxiety and there were no statistically significant differences between groups at baseline measurement. For instance, 20 or 30 self-reported anxiety levels on a VAS from 0 to 100 during the VR neutral environment did not represent significant levels of anxiety. The fact that the SD group had slightly lower anxiety levels in VR alcohol-related environments indicated that these participants did not associate alcohol-related stimuli with the aversive consequences of drinking patterns. However, in our examination of the effectiveness of the ALCO-VR, we found significant differences between AUD and SD groups in anxiety and craving responses across all VR alcohol-related environments. Interestingly, in terms of cue-induced anxiety and craving responses, AUD patients reported similar levels of craving and anxiety during exposure to the VR alcohol-related environments. However, SD cue-induced anxiety responses were lower than their craving responses. SD had a different response tendency from the clinical group regarding anxiety levels. Cue-induced anxiety responses better differentiated the AUD patients and the SD group than craving. It should be noted that craving is elicited by stimuli in both groups, but it may not be associated with anxiety in SD, while, due to the ambivalence of patients in treatment (want to drink and want to remain abstinent) the same cravings may lead to high levels of anxiety.

Addressing age and gender imbalance in this study, our results confirm that AUD patients self-reported greater anxiety and alcohol craving levels compared to participants from the SD group. Similar results were found in a previous study in patients diagnosed with bulimia nervosa (BN), in which anxiety responses discriminated best between BN patients and healthy participants [42]. Although BN and AUD are apparently different disorders, we consider these similar results as an indicator that BN and AUD share common causal and maintenance mechanisms [55].

Apart from the craving and anxiety responses self-reported on the VAS-C and VAS-A respectively, our participants completed the following instruments: AUDIT, MACS, STAI, and MACS-VR. The results showed significantly higher scores in all the instruments in the clinical group than in the control group. The strong correlation between the results of these questionnaires and the scores reported on the VAS-C and the VAS-A confirms the validity of the ALCO-VR software for measuring cue-induced alcohol craving and anxiety, and so we consider that ALCO-VR software may be a useful EMA tool to assess craving and anxiety levels. Interestingly, the MACS-VR instrument exploring alcohol craving immediately after ALCO-VR exposure indicated higher levels of cue-induced alcohol craving than the MACS instrument exploring alcohol craving in the week prior to the experiment. This confirms that the ALCO-VR software can elicit momentary alcohol craving not only in AUD patients, but in SD as well. Therefore, we argue that the ALCO-VR software is a valid instrument for exploring cue-induced anxiety and alcohol craving responses and can differentiate between groups of AUD patients and SD.

The current study is part of a larger project aiming to develop a VR therapy tool based on the cue-exposure paradigm to alcohol-related cues and contexts in patients with severe AUD and with several failed attempts to cease alcohol use. First, we conducted a pilot study testing a VR software based on alcohol-related cues and environments; the results indicated that the software distinguished between heavy drinkers (HD) and light drinkers (LD) in terms of behavioral parameters. LD displayed a preference for non-alcoholic drinks, whereas most heavy drinking individuals preferred alcoholic beverages [56]. In view of these results, we attempted to develop a new VR platform designed to treat patients diagnosed with AUD who were considered “resistant-to-treatment-as-usual” (TAU). A common procedure for identifying specific triggers for alcohol craving is to conduct exhaustive interviews with relevant populations, in our case, with patients with AUD. Therefore, we conducted a second study in which we developed an ad-hoc questionnaire to determine significant factors such as cues and contexts that elicit craving in AUD patients with the aim of creating naturalistic real-life environments [49]. We developed the ALCO-VR software based on the results of the previous study. In addition, the current study is the preceding step to test the efficacy of the cue-exposure therapy (CET) approach using the VR technology in AUD patients.

As substance use disorders and eating disorders resemble addictive behavior mechanisms [57], our study was based on a previous study centered on patients diagnosed with BN, which was conducted at VR-Psy Lab from the University of Barcelona. The aim of the study was to test the efficacy of a VR-cue exposure therapy (VR-CET) for patients diagnosed with BN. The study showed positive results in terms of abstinence rates, anxiety, and craving levels in patients with BN in the VR-CET group compared to the control group. The first step was to perform exhaustive interviews with BN patients to determine triggering factors for binge eating behaviors [58]. Based on the results of that study, VR environments were created and tested in BN patients to examine the elicitation of food craving [42]. These two studies were essential to develop a VR-CET for BN patients. The efficacy of the VR-based therapy software was tested in a multi-site clinical trial and it was found to achieve better outcomes than cognitive-behavioral therapy (CBT) both at the post-treatment assessment [59] and at six-month follow-up [55]. Several limitations should be noted with regard to our study. First, our control group consisted mostly of young women from the Faculty of Psychology, where the majority of students are female. Second, our clinical and control samples were limited. For future studies, we propose that larger samples should be used and that the effectiveness of the ALCO-VR software to differentiate between patients with long-term versus short-term abstinence period in terms of alcohol craving and anxiety should be tested. In addition, to address the gender and age imbalance between our groups, we will consider these limitations for upcoming studies of the same project.

## 5. Conclusions

The current study demonstrated the validity of the ALCO-VR software to induce alcohol craving and anxiety responses, particularly in patients diagnosed with AUD. The ALCO-VR can perform the ecological assessment of momentary cue-induced alcohol craving and anxiety levels, and can differentiate between individuals with AUD and those that are SD.

Considering the promising results of this study, the next step would be to test the therapeutic use of the software in patients diagnosed with AUD. The ALCO-VR is currently being implemented in a multi-site clinical trial testing its efficacy as a cue-exposure therapy tool for patients diagnosed with AUD, who are considered resistant to TAU. The ultimate goal of the project is to reduce relapse rates upon completion of treatment, since relapse remains one of the greatest challenges in AUD treatment and follow-up.

## Figures and Tables

**Table 1:** Data of the self-reported scales.

Scales	Alcohol Use Disorder (AUD) Patients M (SD)	Social Drinkers M (SD)
Alcohol Use Disorder Identification Test (AUDIT)	23.77 (13)	4.5 (2.2)
State-Trait Anxiety Inventory (STAI) (trait part)	34.85 (12)	19 (13)
Multidimensional Alcohol Craving Scale (MACS)	29.23 (9.3)	20 (4.1)
STAI (state part)	18.77 (12.4)	15.64 (14.41)
MACS-VR	36 (12.17)	26.64 (10.9)

**Table 2:** Differences in anxiety and alcohol craving self-reports on VAS-A and VAS-C.

AUD Patients (*N* = 13)				Social Drinkers (*N* = 14)			
	Mean (SD)	Median	IQR ^a^	Mean (SD)	Median	IQR	Z ^b^
Neutral env ^c^							
Anxiety	18.85 (24.54)	5.00	40	32.21 (26.45)	34.00	50	−1.347
Craving	7.69 (16.05)	0.00	7	21.64 (26.38)	11.00	32	−1.726
At home							
Anxiety	54.54 (24.97)	54.00	36	8.21 (11.82)	.50	15	−4.064 ***
Craving	54.77 (22.86)	62.00	33	24.93 (18.71)	19.00	33	−3.180 ***
Bar							
Anxiety	55.69 (25.70)	52.00	43	11.21 (15.55)	2.50	22	−3.980 ***
Craving	58.62 (23.11)	61.00	44	31.64 (21.57)	32.00	42	−2.719 *
Restaurant							
Anxiety	51.77 (20.17)	53.00	41	9.93 (12.17)	5.50	17	−4.162 ***
Craving	57.54 (22.74)	59.00	44	30.93 (20.58)	34.50	39	−2.671 *
Pub							
Anxiety	57.15 (25.32)	54.00	46	12.86 (16.61)	5.00	27	−3.896 ***
Craving	61.85 (24.46)	64.00	50	35.64 (24.48)	39.00	46	−2.258 *

^a^ IQR, interquartile range; ^b^ Mann–Whitney U test. Bonferroni adjustment for multiple testing; *** *p* < 0.001; * *p* < 0.05; ^c^ env, environment.
